# Derived electromyography is an accurate measure of shivering burden during targeted temperature management

**DOI:** 10.1186/cc13687

**Published:** 2014-03-17

**Authors:** T May, DS Seder, GF Fraser, BM McCrum, CF Hoover, RR Riker

**Affiliations:** 1 Maine Medical Center, Portland, ME, USA

## Introduction

Shivering complicates targeted temperature management (TTM) by increasing metabolism, oxygen consumption, resting energy expenditure, and carbon dioxide production and is associated with lower brain tissue oxygen levels; all of these may limit the effectiveness of TTM. However, the recognition and measurement of shivering are subjective and ill-defined. The Bedside Shivering Assessment Scale (BSAS) is the only validated tool to describe the intensity of shivering. We hypothesized that the derived electromyography (dEMG) value measured by the bispectral index monitor (BIS) would agree with energy expenditure due to shivering, compared with the BSAS.

## Methods

We measured continuous indirect calorimetry during a 2 to 5 hour time span during targeted temperature management in 12 patients being treated for hypoxic ischemic encephalopathy after cardiac arrest. Patients were excluded if seizing, requiring >FiO_2 _0.5, exhibiting early spasticity, or not shivering. The BSAS was measured every 15 minutes by a blinded observer and shivering was treated for a BSAS ≥1, as per institutional protocol. The association of dEMG and BSAS as a predictor of resting energy exposure (REE) was measured using linear regression and Pearson's correlation.

## Results

The study population included 12 patients. Average age was 54 years, nine patients were male, eight patients had a CPC of 1 to 3 on hospital discharge. There were a total of 182 measurements of BSAS, dEMG, and REE. There is improved correlation between REE and dEMG compared with REE and BSAS (0.24 (CI = 0.10 to 0.37) vs. 0.10 (CI = -0.04 to 0.24); *P *< 0.001 vs. 0.14). Each increase in dEMG resulted in an increase of 14 kcal of energy expenditure (*P *= 0.003).

## Conclusion

Continuous dEMG power measured by Covidien BIS monitors is a more accurate and less subjective measure of shivering burden compared with the intermittent BSAS. Introducing dEMG into clinical practice may improve recognition of shivering, allow quantification of the metabolic cost of shivering, and serve as a more reliable research tool for diagnostic and treatment strategies of shivering.

